# Congenital Hyperinsulinism in China: A Review of Chinese Literature Over the Past 15 Years

**DOI:** 10.4274/jcrpe.3934

**Published:** 2017-09-01

**Authors:** Wei-Yan Wang, Yi Sun, Wen-Ting Zhao, Tai Wu, Liang Wang, Tian-Ming Yuan, Hui-Min Yu

**Affiliations:** 1 Zhejiang University School of Medicine, Children’s Hospital, Clinic of Neonates, Hangzhou, China; 2 Zhejiang Cancer Hospital, Clinic of Chest Surgery, Hangzhou, China

**Keywords:** congenital hyperinsulinism, Neonate, clinical presentation, Gene mutation

## Abstract

**Objective:**

Congenital hyperinsulinism (CHI) is a rare but severe cause of hypoglycemia. The present study investigates the clinical presentation, therapeutic outcomes and genetic mutations of CHI in Chinese individuals over the past 15 years.

**Methods:**

The authors retrospectively reviewed one case in their department and 206 cases reported from January 2002 to October 2016 in China. PubMed, Ovid Medline, Springer and Wanfang Database, CBMD database, and CKNI database were the sources used to collect the data.

**Results:**

In total, 207 cases were recruited. Of these, the ages of 100 (48.3%) were within the 4^th^ week after birth. Seventy-seven cases (37.2%) were born large for gestational age (LGA). Seizures occurred in 140 cases (67.6%). Among 140 cases (67.6%) who were administered diazoxide treatment, 90 (64.3%) were responsive. Seven cases (3.4%) received octreotide treatment and 19 cases (9.2%) underwent surgery. 63/129 cases (48.8%) were detected to have gene mutations, including ABCC8 (69.8%), KCNJ11 (12.7%), GLUD1, GCK, HADH, and HNF4A. Among the diazoxide-unresponsive cases, gene mutations were detected in 20/36 (55.6%) cases with ABCC8 and in 2 (5.6%) cases with KCNJ11. Among the diazoxide-responsive cases, gene mutations were detected in 8 patients with ABCC8, 4 with KCNJ11, 5 with GLUD1, and 1 with GCK.

**Conclusion:**

The present study indicates that most CHI cases occurred in neonates and that 1/3 of the cases were born LGA. ABCC8 and KCNJ11 are the most common gene mutations. More than half of the diazoxide-unresponsive CHI detected mutations are in ABCC8 and KCNJ11 genes. The GLUD1 gene mutations cause diazoxide-responsive CHI. Identifying the gene mutations can assist in the diagnosis and treatment of CHI.

**What is already known on this topic?**

Several studies have already summarized the clinical and genetic characteristics of congenital hyperinsulinism (CHI) in Beijing and Shanghai cities.

**What this study adds?**

Studies throughout China are still scarce. Our article reviewed the clinical presentation, therapeutic outcomes, and genetic mutations of CHI in the Chinese population over the past 15 years and also compared the CHI in China with that in other countries.

## INTRODUCTION

Congenital hyperinsulinism (CHI) is due to inappropriate insulin secretion in the course of hypoglycemia (1). It is the most common cause of severe and persistent hypoglycemia in newborns and infants. The incidence of CHI is reported to be 1/50.000 live births in a random mating population; however, it can be as high as 1/2500 in communities with high rates of consanguinity (2,3). The clinical presentation of CHI is heterogeneous and varies by age. The severity of hypoglycemia varies from asymptomatic hypoglycemia revealed by a routine blood glucose test to a state of serious hypoglycemic coma or seizures. Major clinical manifestations of CHI reported by other studies include macrosomia, large for gestational age (LGA), seizures, cyanosis, food refusal, lethargy, hypoglycemia, and atypical facial appearance including high forehead with a thin upper lip. Although the clinical symptoms of CHI can reflect the severity to some extent, they serve little to aid the clinicians in the selection of regimen, which is usually correlated with the histopathology of CHI.

Histologically, there are focal and diffuse forms of conditions leading to CHI (4). Focal lesions, which account for approximately 40-50% of all cases, require a partial pancreatectomy. This form typically occurs during infancy rather than in older children. On the other hand, a similar number of cases present with a dysfunctional ATP sensitive potassium (K_ATP_) channel involving the entire pancreas which leads to severe hypoglycemia, requiring a near total pancreatectomy (5). The clinical presentations and prognosis of patients with CHI depend primarily on the histopathology of the pancreas. Most patients with focal forms recover well after surgery, while some diffuse forms show persistent hypoglycemia even after surgery. Thus, it is necessary to differentiate the two forms. However, the clinical symptoms of CHI are non-specific, and thus, additional tools are essential in distinguishing the focal lesions from diffuse forms.

In recent years, the understanding of the genetic mechanisms of CHI has made progress. Mutations in 11 genes, including ABCC8, KCNJ11, GLUD1, GCK, HADH, UCP2, SLC16A1 (MCT1), HNF4A, HNF1A (6), HK1 (7), and PGM1 (8) are known to cause CHI. Among them, the most common and most severe forms of CHI are speculated to be associated with the mutations of the K_ATP_ channel genes (ABCC8 and KCNJ11), encoding the sulfonylurea receptor 1 (SUR1) and Kir6.2 subunits, respectively. Fournet and Junien (9) demonstrated that in about 50% of CHI cases, recessive mutations in the K_ATP_ channel genes cause a diffuse pathology that necessitates a near total pancreatectomy, whereas the loss of heterozygosity together with the inheritance of a paternal mutation causes focal lesion that needs a partial pancreatectomy. Thus, the gene mutation type could assist in diagnosing, differentiating, and identifying the histological type of CHI and in treating the disease.

Though the disease is uncommon, the hypoglycemia caused by CHI can sometimes be extremely severe, frequently leading to severe neurological damage or even death in infancy. Therefore, it is important to diagnose and treat these infants with CHI at the earliest to alleviate the degree of brain damage. Several studies (10,11,12,13) have already summarized the clinical and genetic characteristics of CHI in Beijing and Shanghai; however, studies throughout China are yet lacking. In this article, we aimed to review the present knowledge on the clinical presentation, therapeutic outcomes, and genetic mutations of CHI in the Chinese population and to compare this knowledge with that in other countries.

## METHODS

The search for case reports and case series on confirmed cases of CHI between January 2002 and October 2016 from PubMed, Ovid Medline, Springer and Wanfang Database, CBMD database, and CKNI database retrieved 19 case reports and case series published in core Chinese journals and 4 in journals publishing in English. Three series that summed up the earlier case reports, 4 case reports in Chinese, and 1 case in English were excluded to avoid overlapping. Thus, 12 articles in Chinese and 3 in English, encompassing 207 patients (including 1 case from our department) were included in the survey. The clinical presentation, therapeutic outcomes and genetic mutations of all these 207 CHI patients were analyzed.

A low level of fasting hypoglycemia (<2.8 mmol/L) requiring a high rate of IV glucose infusion (>8 mg/kg/min) to maintain a normal blood glucose level, presence of a detectable insulin level during hypoglycemia, a glycemic response to glucagon injection, undetectable fatty acid and ketone levels and a normal or increased serum ammonia level constituted the diagnostic criteria for CHI (14).

## RESULTS

A total of 207 cases, 1 case from our own department together with the 206 cases of CHI reported previously from China (11,12,13,15,16,17,18,19,20,21,22,23,24,25,26) were available for analysis. Among them, 154 cases were from Beijing city, 32 from Shanghai city, 14 from Guangdong province, 4 from Zhejiang province, and 1 case each from Hunan, Sichuan, and Shandong provinces.

As shown in Table 1, of the 207 cases, 157 (75.8%) were less than 1-year-old. Among these, 100 cases (48.3%) were only 4 weeks old. The patients were males in 114 cases (55%), females in 93 cases (45%), and the male-to-female ratio was 1.23:1. The birth weight of the series ranged from 1.9 to 5.8 kg. Seizures occurred in 140 cases (67.6%), whereas other symptoms such as cyanosis, food refusal, and lethargy were reported in 67 cases (32.3%). Seventy-seven out of 207 (37.2%) cases were born LGA.

Blood tests showed that the blood glucose level of all cases was <2.8 mmol/L and the lowest glucose level reported was 0.3 mmol/L. High levels of insulin (2.4-220 μlU/mL) were observed in all patients. High levels of ammonia (17-128 mmol/L) were detected among a subgroup of cases. Only 1 case was found with focal lesions in the head of pancreas using F-dihydroxyphenylalanine positron emission tomography/computed tomography (F-DOPA PET/CT).

In this series, of the 140 cases (67.6%) who were treated with oral diazoxide, 90 cases (64.3%) reached a normal blood glucose level before discharge from the hospital. Only 7 cases (3.4%) received octreotide treatment, and 4 of these 7 cases (57.1%) reached a normal blood glucose level. Nineteen cases (9.2%) underwent surgery, and in 14 of these 19 cases (73.7%), blood glucose reached a normal level.

**Figure 1:**
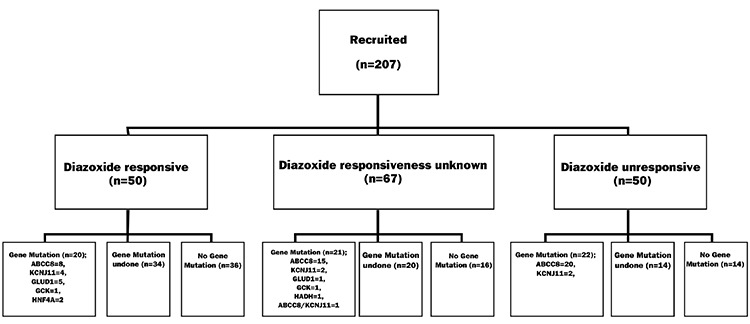
Gene mutation distribution of 63 cases detected in 129 cases

One hundred-twenty nine cases underwent gene mutation test, and 63 of these cases (48.8%) showed mutations in ABCC8, KCNJ11, GLUD1, GCK, HADH, and HNF4A genes. Two of these cases were found to be homozygous for ABCC8 mutation (1 case unresponsive to diazoxide, and the other with unknown diazoxide response). Three cases were found to be compound heterozygous for ABCC8 mutation (2 cases diazoxide-responsive, 1 case with unknown diazoxide response), 15 cases were found to have heterozygous mutation in ABCC8 gene (9 cases diazoxide-unresponsive, 4 cases diazoxide-responsive, and 2 cases with unknown diazoxide responsiveness). Two cases were found to possess heterozygous mutation in KCNJ11 gene (1 case diazoxide-unresponsive, the other one diazoxide-responsive); 1 case was found to have heterozygous mutation in GLUD1 gene (diazoxide-responsive), 8 cases had de novo mutations (5 ABCC8, 2 GLUD1, 1 GCK), and 32 cases were not analyzed for the parents’ gene mutation test (Figure 1).

In this series, 63 cases were detected to possess 60 gene mutations, including 39 mutations in ABCC8 gene, 8 in KCNJ11, 6 in GLUD1, 2 in GCK, 3 in HADH, and 2 mutations in HNF4A gene. Mutations in ABCC8 gene were detected in 8 cases; one mutation in KCNJ11 gene and one in GLUD1 gene appeared twice.

We recruited 90 cases who went through gene mutation test and received diazoxide treatment. Among them, 54 cases were responsive to diazoxide and 20 were detected to have positive gene mutations. These mutations were as follows: in ABCC8 gene-8 cases, in KCNJ11-4, in GLUD1-5, in HNF4A-2, and in GCK gene-1 case (Table 2). The other 36 cases were unresponsive to diazoxide treatment, and 22 cases were detected to have positive gene mutation. These gene mutations were in ABCC8 gene in 20/36 cases (55.6%) and in KCNJ11gene in 2 cases (5.6%) (Table 3).

## DISCUSSION

Most of the patients in the series were the offspring of families who resided in the eastern part of China. The vast majority of CHI cases were from Beijing and Shanghai cities and Guangdong and Zhejiang provinces, though a small number of cases were also reported from other regions such as Hunan, Sichuan, and Shandong provinces. To our knowledge, this is the first report involving the distribution of CHI. We speculated that this geographic disequilibrium was partially due to the disequilibrium of the economy and to missed cases due to ignorance and misdiagnosis of this disease in different areas of China related to social, economic factors. Possibly, the medical personnel in those areas did not realize the significance of identifying the cause of persistent hypoglycemia and did not attempt to investigate the gene mutation status in these patients.

In our study, 48.3% of cases were found to present clinical manifestation of CHI within 4 weeks after birth. This finding was similar to the results of a Taiwanese study with 46% of cases with neonatal onset (27). Our study also demonstrates that macrosomia can often be observed among the patients, and LGA accounts for 37.2%. We speculate that the underlying reason for macrosomia should be ascribed to be the severe prenatal hyperinsulinism. A study comprising 114 patients (28) revealed that 27% of patients with neonatal-onset CHI had a birth-weight SDS of >2. Shen et al (22) reported 15 neonatal cases of CHI, of which 10 cases were LGA and blood glucose level returned to normal in only 3 patients in this series. Thus, we propose that an early-onset age of CHI together with a high birth weight are indicators of severity of this condition.

In addition, our findings demonstrate that seizures frequently occurred in CHI, as reported by Ferry et al (29) and that cyanosis, food refusal, lethargy, and hypoglycemia are also features of CHI. The clinical presentation is nonspecific, and the conventional ultrasound scan, computed tomography, and magnetic resonance imaging could not detect any lesion.

The findings of this study show that diazoxide is the first-line treatment in this condition. The positive response rate (64.3%) to diazoxide in our sample is in accordance with that of 66% in 175 cases of CHI reported by de Lonlay et al (30). We recruited only 7 cases receiving octreotide treatment, 4 of these cases were responsive. Although octreotide treatment could be used in diazoxide-unresponsive cases, it could cause cholestasis and hepatitis (31,32). Elevated levels of liver enzymes and gallbladder pathology were detected in the patients treated. Octreotide treatment may also impose an enormous economic burden on the family, thereby, limiting its clinical usage. 

Surgery is the last option for drug-resistant CHI patients. In our review, 19 such cases underwent the operation and 14 of these cases were restored to normal glucose level after surgery. However, since F-DOPA PET/CT is not available in Mainland China, most patients are obliged to undergo a subtotal pancreatectomy and endure the risk of becoming a diabetic as a side-effect.

Nevertheless, now that gene mutation test is available, several researchers are focusing on the relationship between diazoxide treatment and gene mutation type. Previously, the reasons for the differences in responsiveness to diazoxide treatment were unknown and were ascribed by some researchers to different histological patterns of CHI (33). Snider et al (6) reported that 91% of diazoxide-unresponsive cases were correlated with recessive KATP channel gene mutations, while 41% of diazoxide-responsive cases were correlated with dominant K_ATP_ mutations.

In our study, 129 cases underwent gene mutation test and 48.8% of these cases were detected to have a gene mutation, which is a much lower frequency than that reported by Park et al (33). These authors reported a frequency of 82% as mutation findings in a Korean population. However, our results are similar to those of other researchers who reported a frequency of 45.3% mutations in CHI patients (34). These differences in frequency may be partially due to differences in ethnicity-related genetics. Moreover, Park et al (33) recruited only 17 patients, and thus, a large-scale cohort is essential to illustrate the frequency in a population. Among the 63 cases of gene mutation detected in our series, the KATP channel gene mutations (ABCC8 or KCNJ11) account for 82.5% of the gene mutations; this result is similar to the 84.2% frequency reported by Yorifuji et al (34) and 80.1% by Kapoor et al (35).

We detected 60 gene mutations out of 63 cases in the Chinese population, with 8 ABCC8, 1 KCNJ11, and 1 GLUD1 gene mutations appearing twice, and 2 ABCC8 gene mutations appearing three times. This is the first report to sum up the occurrence rate of gene mutations in a population and we hope these findings will facilitate the clinicians’ work in CHI cases.

Our results indicate that all GLUD1 gene mutations contribute to diazoxide responsiveness. This finding is in accordance with the study by Snider et al (6). Some cases with a heterozygous ABCC8/KCNJ11 mutation are also diazoxide-responsive, which is similar to the result of Kapoor et al (35). GCK mutation may also be attributed to diazoxide-responsive CHI; in most cases it is medically responsive, although in some cases, surgery may be required (36). Two HNF4A gene mutation cases were diazoxide-responsive in our study; Kapoor et al (37) also elucidated that CHI patients with HNF4A can exhibit mild, transient to severe, persistent hypoglycemia and are diazoxide-responsive.

We also demonstrated that the cases with compound heterozygous recessively acting ABCC8/KCNJ11 mutations, or homozygous ABCC8/KCNJ11 recessively acting mutation, or some cases with a heterozygous ABCC8/KCNJ11 mutation may show diazoxide-unresponsiveness, which is in accordance with the results of Kapoor et al (35).

In our study, one ABCC8 gene mutation (c.331G>A; p.G111R) occurred twice, but one case was responsive to diazoxide treatment, while the other was unresponsive. The reason for this difference in response is unknown, but we see that the responsive case had a paternally inherited heterozygous mutation, while a gene mutation test was not performed in the parents of the unresponsive case. It can be speculated that the unresponsive case may have had a paternally inherited mutation and loss of heterozygosity that caused focal CHI.

Nowadays, the gene mutation test can be conducted easily with rapid results in some developed countries. However, in China, the cost of gene mutation test is high and it takes a long time to obtain the results. One of the aims of this study was to facilitate the diagnosis and treatment of CHI by the clinician in China, where F-DOPA PET/CT is not available. Some gene mutations appearing two or three times may indicate their frequent occurrence in the Chinese population.

In conclusion, our study suggests that nearly half of CHI cases occur in neonates, and the most common symptom is seizures. The first-line treatment of CHI is diazoxide treatment, octreotide is not used often, and surgery is the option due to drug unresponsiveness. F-DOPA PET/CT is not available in Mainland China. In Chinese patients, ABCC8 and KCNJ11 are the most common gene mutations, and GLUD1 ranks second. Half of the gene mutations of diazoxide-unresponsive CHI are KATP (ABCC8 and KCNJ11) mutations, homozygous or compound heterozygous mutations, and some are a heterozygous ABCC8 mutation. GLUD1 always caused diazoxide-responsive CHI.

CHI is a complex disorder with non-specific presentations. Our study suggests that gene mutation test is now performed more frequently in China, although not as often as in other developed countries. Obtaining rapid results in genetic testing is uniquely valuable for CHI patients in China.

## Figures and Tables

**Table 1:**
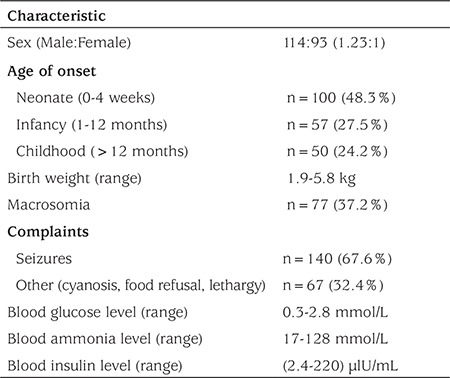
Clinical characteristics of congenital hyperinsulinism in Chinese patients

**Table 2:**
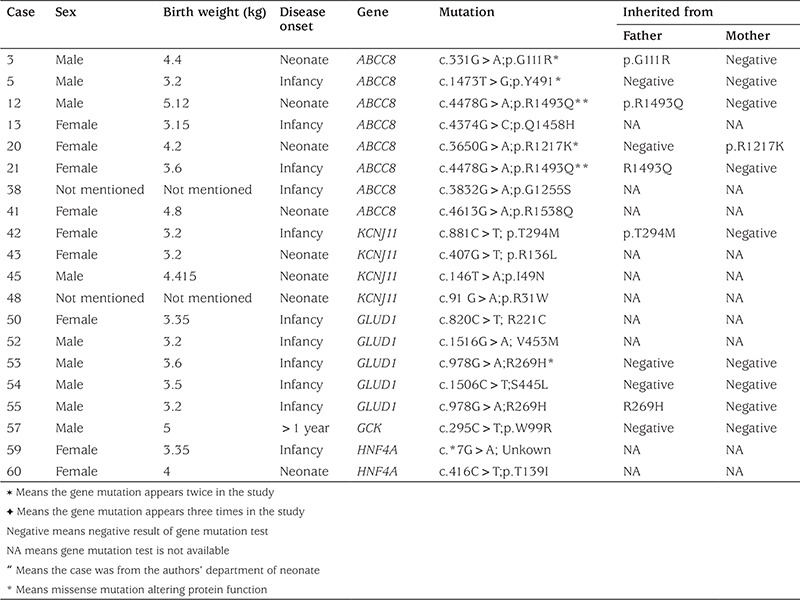
Diazoxide-responsive congenital hyperinsulinism cases and gene mutations

**Table 3:**
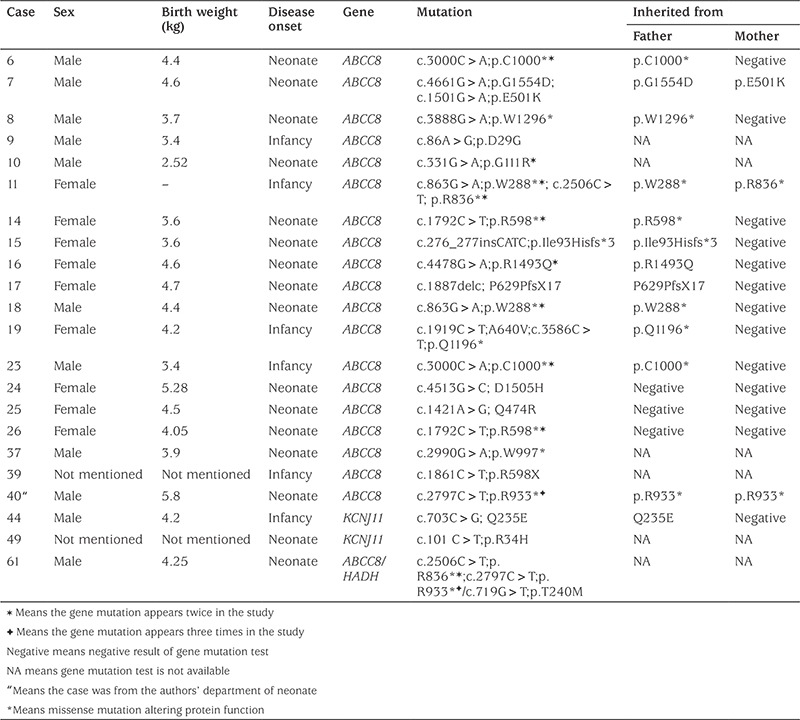
Diazoxide-unresponsive congenital hyperinsulinism cases and gene mutations
